# High Intensity Training Improves Health and Physical Function in Middle Aged Adults

**DOI:** 10.3390/biology3020333

**Published:** 2014-05-12

**Authors:** Simon Adamson, Ross Lorimer, James N. Cobley, Ray Lloyd, John Babraj

**Affiliations:** 1 Division of Sport and Exercise Science, Abertay University, Dundee, Scotland, DD1 1HG, UK; E-Mails: s.adamson@abertay.ac.uk (S.A.); ross.lorimer@abertay.ac.uk (R.L.); j.cobley@abertay.ac.uk (J.N.C.); 2 Leeds Trinity University, Leeds, LS18 5HD, UK; E-Mail: ray.lloyd@leedstrinity.ac.uk

**Keywords:** VO_2_ peak, oral glucose tolerance, functional capacity, middle age

## Abstract

High intensity training (HIT) is effective at improving health; however, it is unknown whether HIT also improves physical function. This study aimed to determine whether HIT improves metabolic health and physical function in untrained middle aged individuals. Fourteen (three male and eleven female) untrained individuals were recruited (control group n = 6: age 42 ± 8 y, weight 64 ± 10 kg, BMI 24 ± 2 kg·m^−2^ or HIT group n = 8: age 43 ± 8 y, weight 80 ± 8 kg, BMI 29 ± 5 kg·m^−2^). Training was performed twice weekly, consisting of 10 × 6-second sprints with a one minute recovery between each sprint. Metabolic health (oral glucose tolerance test), aerobic capacity (incremental time to exhaustion on a cycle ergometer) and physical function (get up and go test, sit to stand test and loaded 50 m walk) were determined before and after training. Following eight weeks of HIT there was a significant improvement in aerobic capacity (8% increase in VO_2_ peak; *p* < 0.001), physical function (11%–27% respectively; *p* < 0.05) and a reduction in blood glucose area under the curve (6% reduction; *p* < 0.05). This study demonstrates for the first time the potential of HIT as a training intervention to improve skeletal muscle function and glucose clearance as we age.

## 1. Introduction

Middle age (defined as 35 to 58 years old) is associated with a loss of aerobic capacity, as evidenced by a decline in VO_2_ max of approximately 8% per decade [1] and an associated functional decline of 15% between young and middle age [2]. This loss of skeletal muscle performance/skeletal muscle endurance capacity is associated with a loss of mitochondrial enzyme activity and impaired mitochondrial protein synthesis at rest [3]. Further there is a loss of insulin sensitivity of 8% per decade in both males and females [1]. Together the loss of skeletal muscle performance is associated with an increased risk of life style diseases such as type 2 diabetes (T2D) and cardiovascular disease during middle age.

Exercise is a powerful therapy for treatment and prevention of T2D and other chronic disease such as cardiovascular disease [4]. It has been shown that physical activity alone, or in combination with dietary changes, reduces the risk of developing T2D in populations with impaired glucose tolerance by >50% [5,6]. Endurance training has been shown to increase both insulin sensitivity and mitochondrial function in mixed gender middle age populations [1,7]. Exercise intensity determines the magnitude of these improvements with higher intensities resulting in greater increases in cardio-respiratory fitness and insulin sensitivity post-training [8,9]. Despite the well-documented health benefits of exercise only 40% of men and 28% of women in the UK achieve the recommended 30 minutes of moderate intensity exercise on five days of the week [10]. Lack of time to exercise, due to work or family commitments, is cited as the most common reason given for not participating in the general population [11] and in middle aged females [12]. This suggests that time efficient training paradigms may be a more effective way to reduce disease risk in middle age populations and promote long term healthy ageing.

High intensity training (HIT) has been shown to be a time efficient exercise paradigm that improves exercise performance to the same extent as traditional endurance training [13] and increases skeletal muscle GLUT4 concentration [14]. Utilising the same progressive training protocol of Burgomaster *et al.*, Babraj *et al.* demonstrated that two weeks of HIT improves insulin sensitivity in young sedentary males [15]. Metcalfe *et al*. have recently reported improved insulin sensitivity in young men following six weeks of HIT training involving three sessions per week of 2 × 20 second sprints against 7.5% of bodyweight [16]. However the study found no improvement in blood glucose control or insulin sensitivity in young female participants. This is in contrast to Richards *et al.* who demonstrate improved insulin sensitivity in a mixed young population [17]. In response to a whole body Tabata high intensity interval training stimulus female participants see a significant change in cardiovascular fitness and muscular endurance [18]. The overall intensity of the exercise session has been shown to be most important for the acute response to sprint interval cycling [19]. Together this suggests that 7.5% bodyweight cycle sprints may be too heavy for female participants leading to a different training stimulus.

The purpose of this study was to determine whether shorter duration HIT involving six second sprints and totalling 60 seconds of exercise per session, performed twice a week could elicit improvements in cardio-metabolic health and physical function outcomes in an untrained middle age population. It was hypothesized that eight weeks of extremely short duration HIT training would improve health outcomes and functional capacity.

## 2. Experimental Section

### 2.1. Participants

Fourteen (three male and eleven female) untrained individuals, who self-reported baseline physical activity levels as having not participated in a regular training regime for at least 12 months prior to the study, were recruited to take part in this study. All participants were healthy and were not taking any prescribed medications. Participants were allocated to either a control (CON, n = 6, 4 participants were overweight as defined by BMI, Table 1) or high intensity training group (HIT, n = 8, all eight participants were overweight as defined by BMI, Table 1) using a 4:2 block randomisation protocol. No power analysis was done prior to the study, but we know from previous studies that a sample size of six participants is enough to see significant improvements in endurance capacity and lactate metabolism when using this exercise protocol [20]. The CON group were asked to maintain their normal activities throughout the study period. There were no significant changes in body weight or blood pressure during the study (Table 1). However, there was a difference in weight between the control and HIT group. The participants were informed of the experimental protocol both verbally and in writing before giving informed consent. The study protocol was approved by the institutional Ethics committee and was conducted in accordance with the Helsinki Declaration.

**Table 1:** Participant characteristics; * *p* < 0.05 pre *versus* post, however, there was no significant difference in the magnitude of the change between groups; † *d* > 0.7 effect size of magnitude of change between the groups.

Parameter	CON		HIT	
	Pre	Post	Pre	Post
Sex	1 male, 5 female		2 male, 6 female	
Age (y)	42 ± 8	42 ± 8	43 ± 8	43 ± 8
Height (cm)	162 ± 7	162 ± 7	165 ± 7	165 ± 7
Weight (kg)	64 ± 9	64 ± 10	80 ± 8	79 ± 9
BMI (kg·m^−2^)	24.3 ± 1.9	24.3 ± 1.6	29.5 ± 4.1	29.1 ± 4.6 †
Energy Intake (Kcal·d^−1^)	4651 ± 1767	4361 ± 1544	5217 ± 1918	5567 ± 1262
Systolic Blood Pressure (mmHg)	128 ± 20	127 ± 11	137 ± 11	133 ± 9
Diastolic Blood Pressure (mmHg)	77 ± 11	78 ± 7	81 ± 9	79 ± 5
Fasting glucose (mmol·L^−1^)	4.3 ± 0.5	4.2 ± 0.5	4.6 ± 0.3	4.3 ± 0.2 *†
2h glucose (mmol·L^−1^)	4.7 ± 0.9	4.5 ± 1.1	5.4 ± 1.3	4.8 ± 1.2 *†

### 2.2. Experimental Protocol

Baseline exercise performance, physical function and health parameters were determined prior to the commencement of the HIT training program. Baseline testing consisted of two sessions separated by at least 48 hours. On the first baseline session height (SECA 264 stadiometer, SECA, Birmingham, UK) and weight (SECA 799 digital scales, SECA, Birmingham, UK) were recorded, and BMI was then calculated. Three day food diaries were collected prior to baseline testing and after eight weeks. Dietary intake did not change over the course of the study (Table 1).

#### 2.2.1. Session 1: Oral Glucose Tolerance Test (OGTT)

Participants were asked to refrain from performing any strenuous exercise activity for two days prior to the OGTT, and attended the laboratory having fasted overnight from 10 pm. Blood glucose samples were collected by finger prick incision, and analysed using a Freestyle Light Blood Glucose Analyser (Abbott Diabetes Care, Maidenhead, UK), before and every 20 minutes for 120 minutes after the ingestion of 75 g of glucose (410 mL of Lucozade Original, GlaxoSmithKline, Brentford, UK).

#### 2.2.2. Session 2: Physical Function

There is a strong relationship between muscle power and physical function in men and women across the lifespan [2,21]. However, tests of physical function are related to activities of daily living [22] which make them more applicable to a non-sporting population. Participants performed three different functional tests in the same order as follows:

##### 2.2.2.1. Get Up and Go

Participants rose from a chair only using their legs, walked forward three metres, turned and returned to the chair then sat back down, with total time taken to complete this recorded [23]. Time was recorded using a Quantum 5500 stop clock (EA Combs Ltd., London, UK) and the test was repeated on three occasions with the average of all three times reported.

##### 2.2.2.2. Sit to Stand

Participants rose from a chair and sat back down as many times as possible in 30 seconds without using their arms, with total number of times they stood up and down recorded [24]. This was repeated on three occasions with the average of all three completions reported. 

##### 2.2.2.3. 50 Metre Loaded Walk

participants walked 50 metres as quickly as possible between two cones spaced 10 metres apart, whilst carrying two canvas tote bags weighted with 12.5% of the participant’s body weight in each bag for males, and 10% of the participant’s body weight in each bag for females [21]. This was repeated on two occasions and total time (recorded using a Quantum 5500 stop clock, EA Combs Ltd., London, UK) to complete the 50 m walk was recorded, with the average of both attempts reported. 

#### 2.2.3. Time to Exhaustion

Participants performed an exhaustive incremental cycling test [25] to determine peak oxygen uptake (VO_2_ peak) via breath by breath analysis (Cortex Metamax system, Birmingham, UK). After cycling against 1 kg of weight for 5 min, the weight of the bike cradle increased by 0.5 kg every 2 min until the participant could no longer maintain a speed of 50 rpm or until volitional exhaustion occurred. Time was recorded using a Quantum 5500 stop clock (EA Combs Ltd., London, UK) and VO_2_ peak was determined as the highest average 10 s value. 

#### 2.2.4. High Intensity Training

The HIT training protocol was similar to that used previously by Jakeman *et al.* [20]. 16 sessions of HIT were spread over an eight week period, with one or two days of rest between each sprint. There was no warm-up or cool down in the training sessions. Each training session consisted of 10 repeated 6-s all-out cycling efforts against 7.5% of body weight for males and 6.5% for females (Monark peak bike Model 894E, Monark Exercise AB, Vansbro, Sweden), added to the bike once the participant was cycling at 100 rpm with 1 min of passive recovery between sprints. Bodyweight percentages were determined due to the difference in muscle mass reported between males and females [26].

#### 2.2.5. Post-Training Assessment

The OGTT, body composition analysis, physical function tests and exercise performance test were repeated, in the same order as before, after the completion of the HIT intervention. On average there were 5 ± 2 days between the final HIT session and post OGTT for both HIT and CON with the other test carried out 24 h later.

#### 2.2.6. Calculations and Statistical Analysis

Glucose area under the curve (AUC) was calculated using the conventional trapezoid rule. Data was checked for skewness and kurtosis and these values did not exceed twice the standard error, therefore the data was deemed to be normally distributed. Differences between pre and post data collections for participant characteristics, exercise performance, functional ability and plasma glucose AUC were compared using the methodology proposed by Hopkins WG for controlled trials [27]. The significance level was set at 0.05 (*p* < 0.05) and the Cohen’s effect size was defined as follows: d < 0.2 trivial effect, 0.2–0.5 small effect, 0.6–1.1 moderate effect and 1.2–1.9 as a large effect [28].

## 3. Results and Discussion

### 3.1. VO_2_ Peak

**Figure 1:**
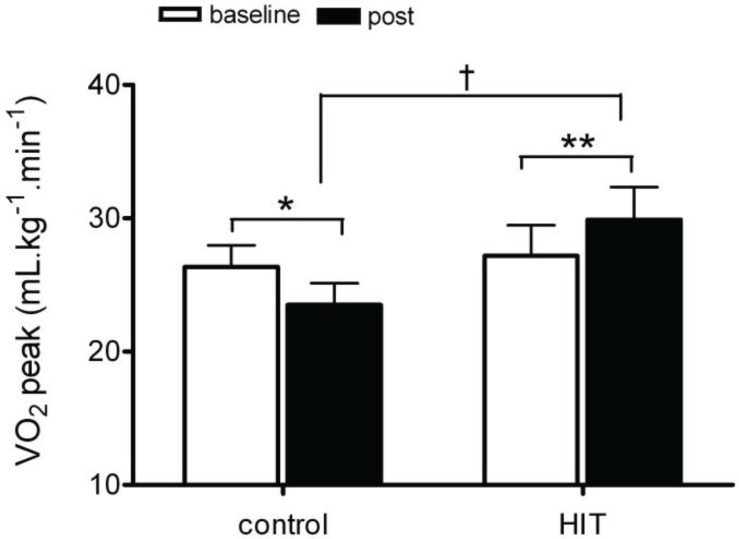
Change in VO_2_ peak for control and HIT pre and post intervention. * *p* < 0.05 pre to post within groups; ** *p* < 0.001 pre to post within groups; † *p* < 0.02 magnitude of change between groups.

At baseline, VO_2_ peak was not significantly different between the two groups (CON group: 26.3 ± 4 mL.kg^−1^·min^−1^; HIT group: 27.2 ± 7 mL.kg^−1^·min^−1^; *p* > 0.05; Figure 1) and was reduced in the control group when repeated after eight weeks (pre: 26.3 ± 4 mL·kg^−1^·min^−1^; post: 23.5 ± 4 mL·kg^−1^·min^−1^; *p* = 0.02; Figure 1). Following eight weeks of HIT training, VO_2_ peak was significantly increased by 8% (pre training 27.2 ± 7 mL·kg^−1^·min^−1^, post training 29.9 ± 7 mL·kg^−1^·min^−1^; *p* = 0.0007; *d* = 1.03; Figure 1). The size of change in VO_2_ peak was significantly greater following HIT (CON group: −2.8 ± 2.0 mL·kg^−1^·min^−1^; HIT group: 2.7 ± 2.5 mL·kg^−1^·min^−1^; *p* = 0.017; *d* = 2.4). These findings are consistent with previous studies investigating the effects of HIT on aerobic performance in younger populations [13,29,30]. VO_2_ peak has been shown to be an independent predictor of cardiovascular disease morbidity and all-cause mortality in an adult population [31]. In a middle aged population there is a decline in VO_2_ peak [1], increasing cardiovascular disease risk. Further there is a positive relationship between VO_2_ peak and endothelial function in non-obese males [32]. The underlying mechanisms for improvement in VO_2_ peak following HIT are poorly understood. Two week HIT interventions have traditionally been associated with improved peripheral adaptations such as increased mitochondrial enzyme activity [13,26]. However recent studies utilising longer duration HIT programs have demonstrated improved cardiovascular and endothelial function [30,33,34,35]. Therefore it seems likely that the current HIT program leads to an improved cardiorespiratory fitness via both central and peripheral adaptations, which will reduce cardiovascular disease risk in a middle aged population.

### 3.2. Functional Capacity

**Figure 2:**
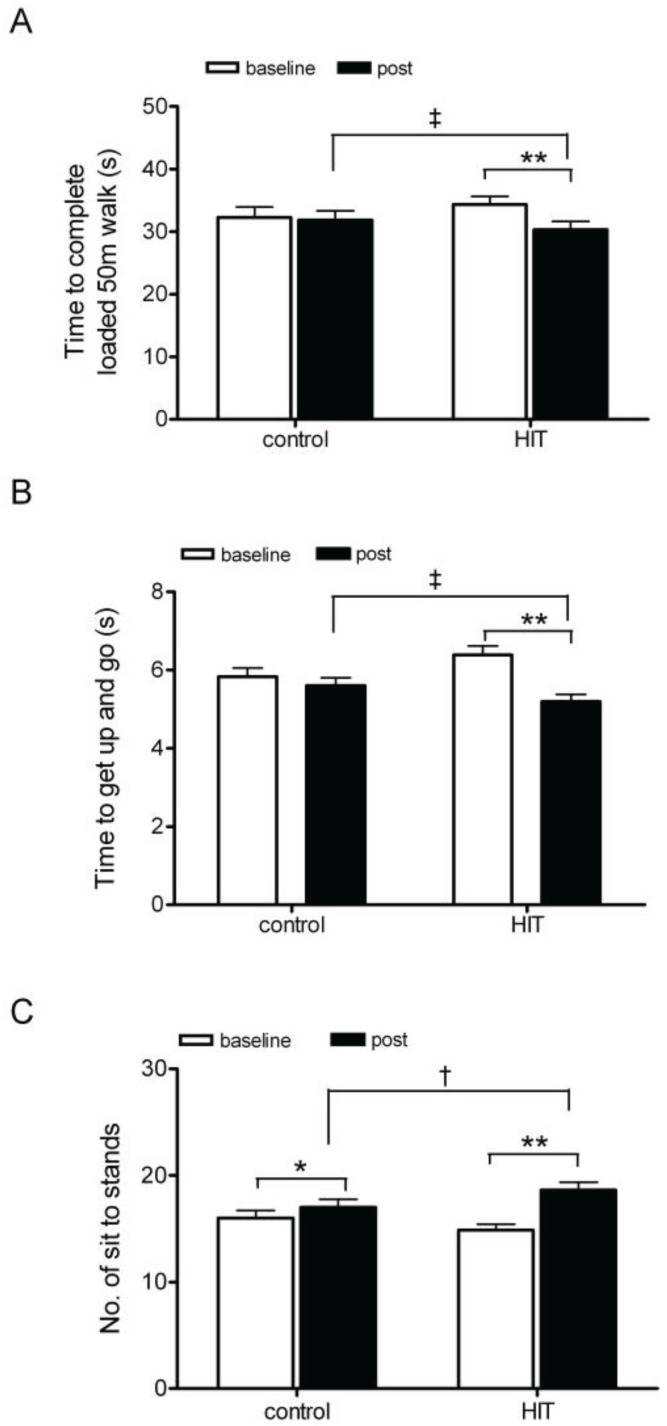
(**a**) Time taken to complete a loaded 50m walk test. (**b**) Time taken to complete a get up and go test. (**c**) Total number of sit to stands performed in 30 seconds. * *p* < 0.05 pre to post within groups; ** *p* < 0.001 pre to post within groups; † *p* < 0.02 magnitude of change between groups; ‡ *p* < 0.001magnitude of change between groups.

At baseline, functional capacity was not significantly different between the two groups (*50 m loaded walk*: CON group 32 ± 4 seconds; HIT group 34 ± 4 seconds; *p* > 0.05; Figure 2A; *Get up and go*: CON group 6 ± 1 seconds; HIT group 6 ± 1 seconds; *p* > 0.05; Figure 2B; *Sit to stand*: CON 16 ± 2 completions, HIT group 15 ± 2 completions; *p* > 0.05; Figure 2C). All functional tests were unchanged in the control group except sit to stand which improved after eight weeks (*50 m loaded walk*: pre 32 ± 4 seconds; post: 32 ± 4 seconds; *p* > 0.05; Figure 2A; *Get up and go*: pre: 6 ± 1 seconds; post: 6 ± 1 seconds; *p* > 0.05; Figure 2B; *Sit to stand*: pre: 16 ± 2 completions; post: 17 ± 2 completions; *p* = 0.01; Figure 2C). Following eight weeks of HIT training, all functional tests were significantly improved (*50 m loaded walk*: pre training 34 ± 4 seconds, post training 30 ± 4 seconds; *p* < 0.0001; *d* = −1.06; Figure 2A; *Get up and go*: pre training 6 ± 1 seconds, post training 5 ± 1 seconds; *p* = 0.001; *d* = −1.98; Figure 2B; *Sit to stand:* pre training 15 ± 2 completions, post training 19 ± 2 completions; *p* = 0.001; *d* = −1.76; Figure 2C). The size of change in functional capacity was significantly in the HIT group compared to the control group (*50 m loaded walk*: CON group: −0.5 ± 0.8 seconds; HIT group: −3.9 ± 1.2 seconds; *p* = 0.0001; *d* = −3.36; *Get up and go*: CON group: −0.2 ± 0.3 seconds; HIT group: −1.2 ± 0.6 seconds; *p* = 0.0001; *d* = −1.86; *Sit to stand*: CON group: 1 ± 1 seconds; HIT group: 4 ± 2 seconds; *p* = 0.003; *d* = 2.09). The underlying mechanism for improvement in functional capacity with HIT still needs to be defined. The loss of physical function in middle age is strongly associated with declining mitochondrial function [36]. HIT has consistently been shown to increase mitochondrial enzyme activity in skeletal muscle regardless of intensity or duration [13,30,36,37]. Alternatively changes in fat metabolism or insulin resistance could drive improvements in physical function [38,39]. HIT has been shown to increase daily energy expenditure [40], whilst we report no increase in calorie intake (Table 1), suggesting greater utilisation of stored fuels after a HIT session. HIT has also been shown to improve skeletal muscle insulin sensitivity after two weeks [15]. Therefore improvements in physical function in the current study are likely to be related to improved muscle mitochondrial function, muscle insulin sensitivity and altered fat metabolism following eight weeks of twice weekly HIT.

### 3.3. Plasma Glucose AUC

**Figure 3:**
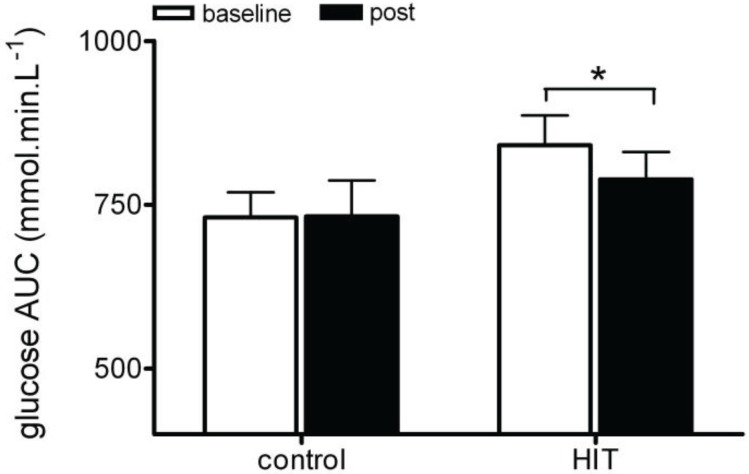
Change in glucose AUC for control and HIT pre and post intervention. * *p* < 0.05 pre to post within groups.

At baseline, there was a difference in bodyweight between the two groups. However, there was no correlation between glucose AUC and bodyweight at baseline (R = 0.1) which suggests that bodyweight is not affecting blood glucose control. Glucose AUC was not significantly different between the two groups (CON group 731 ± 96, HIT group 841 ± 128; *p* > 0.05; Figure 3) and remained unchanged in the control group when repeated after eight weeks (pre: 735 ± 96; post: 732 ± 134; *p* > 0.05; Figure 3). Following eight weeks of HIT training, glucose AUC measured 5 days after the last HIT session was reduced by 6% (pre: 841 ± 128; post: 788 ± 118; *p* = 0.05; *d* = −0.87, Figure 3). The size of change in plasma glucose AUC was not significantly different following HIT, however there was a large effect size (CON group: 1.5 ± 59; HIT group: −52 ± 64; *p* = 0.13; *d* = −0.87). The decrease in glucose AUC was seen five days after the last exercise session in a mixed gender middle aged population. The magnitude of change in glucose AUC is similar to that reported previously in a younger population [15,17]. Skeletal muscle is the major site of insulin mediated blood glucose clearance [41] and the improvements seen in whole body glucose processing may be a result of an increased skeletal muscle GLUT4 content and total glycogen content after HIT [4] due to increased skeletal muscle glycogen utilisation during the training [42]. Alternatively, the improvement in glucose clearance could be due to altered skeletal muscle fat metabolism. High levels of non-esterified fatty acids have been shown to inhibit the skeletal muscle uptake of glucose [43] and HIT has been shown to reduce circulating non-esterified fatty acids [15].

## 4. Conclusions

This study demonstrates for the first time that HIT needs to be performed only twice a week to see major improvements in aerobic capacity, functional capacity and metabolic health in a middle aged population. These findings are in contrast to previous literature where three sessions of HIT per week have been required to improve metabolic health and aerobic capacity up to three days after the last HIT session [15,17,18,29]. We also demonstrate for the first time the potential of HIT as a method of improving and maintaining physical function in untrained middle-aged individuals. Given that time is the major barrier to exercise in a middle aged population [12] then the current exercise guidelines may not be preferred to promote health improvements. Further, the improvements in physical function following HIT can be utilised to reverse functional decline with ageing. This suggests that HIT, lasting no more than 11 minutes, may be used as a time-efficient method of reducing the risk of disease and functional decline as we reach middle age and as little as two sessions per week are required to obtain improvements in health outcomes in a middle aged population. Despite the improvements seen, the sample size in the present study is small and further studies are required to determine population based responses to determine whether there is a genetic limitation in adaptations to this exercise, the effect of fat mass on the magnitude of response and longer duration adaptation to this type of exercise.
